# Insights into how JIP3 and JIP4 adaptor proteins relieve the auto-inhibited form of the kinesin-1 motor

**DOI:** 10.1016/j.jbc.2026.113355

**Published:** 2026-07-22

**Authors:** Fernando Vilela, Mélanie Chenon, Valérie Campanacci, Ania Alik, Christophe Velours, Jessica Andreani, Paola Llinas, Julie Ménétrey

**Affiliations:** Université Paris-Saclay, CEA, CNRS, Institute for Integrative Biology of the Cell (I2BC), Gif-sur-Yvette, France

**Keywords:** JIP3, JIP4, RH1 domain, KIF5, kinesin-1, auto-inhibition

## Abstract

While our understanding of kinesin autoinhibition mechanisms is advancing, key insights into kinesin activation, especially autoinhibition relieved by cargo-motor adaptors, remain unclear. JIP3 and JIP4 are adaptors that activate kinesin-1 and dynein-dynactin motors, enabling bidirectional transport along microtubules. Here, we characterized the interaction between the kinesin-1 heavy chain, KIF5B, and both JIP3 and JIP4 (JIP3/4), using mutagenesis and binding experiments associated with modelling. We identified the minimal regions of JIP3/4 and KIF5B required for interaction. JIP3/4 binds to the CC4 coiled-coil domain of KIF5B, with no significant interaction detected with the distal tail. Conversely, KIF5B-CC4 binds to the RH1 domain of JIP3/4, with the preceding disordered N-terminus modulating the force of the binding. Notably, JIP3 and JIP4 exhibit ∼40-fold differences in affinity for KIF5B. We performed extensive site-directed mutagenesis on KIF5B-binding regions of JIP3/JIP4 targeting accessible charged residues and sequence differences between JIP3 and JIP4. This revealed multiple weak-affinity binding sites that collectively define an extensive KIF5B-binding surface on the RH1 domain of JIP3. Importantly, the KIF5B-binding surface on JIP3 differs from that of the DLIC (dynein light intermediate chain), confirming no competition between the two motors. Altogether, the fragment and mutant mapping of KIF5B binding to JIP3 and JIP4 presented here provides insights into how the auto-inhibited form of kinesin-1 might be relieved, with the CC4 region playing a central role.

Motor–cargo adaptors are critical regulators of intracellular trafficking, not only because they recruit and link motors to cargos for transport, but also because they participate in motor regulation. JIP3 and JIP4 (collectively referred to as JIP3/4) are adaptors that recruit and activate both the dynein–dynactin complex and kinesin-1 motors, enabling bidirectional cargo transport along microtubules (1, 2, 3, 4, 5, 6). Recently, a cryo-EM structural study revealed how the dynein–dynactin complex is recruited by JIP3, providing insights into how this adaptor activates the motor (7). By contrast, only limited structural data are available to understand how JIP3/4 recruit and activate the kinesin-1 heavy chain (KHC).

Kinesin-1 is a heterotetramer composed of a homodimer of kinesin heavy chains (KHCs) associated with two light chains (KLCs). Each KHC consists of an N-terminal motor domain (MD) and neck coil (NC), followed by a long stalk formed by four coiled-coil segments (CC1–CC2–CC3–CC4), and a C-terminal unstructured tail containing the autoinhibitory IAK motif (Fig. 1*A*). In humans, KHC is encoded by three genes, KIF5A, KIF5B, and KIF5C, which share a highly conserved motor domain and stalk, but differ in their C-terminal tail regions. The coiled-coil segments mediate KHC homodimerization and also serve as binding sites for KLCs (primarily CC3 and CC4) (8), as well as for other partners (*e*.*g*., microtubule-associated proteins and motor–cargo adaptors) at CC1 and CC4. In addition, these coiled-coil segments contribute to the formation and stabilization of the autoinhibited conformation of kinesin-1. When not bound to cargo, kinesin-1 is maintained in an inactive, autoinhibited state, preventing ATP hydrolysis and microtubule binding. Recently, 3D models of the folded conformation of KHC (either alone or in complex with KLC) have advanced our understanding of the structural basis of kinesin-1 autoinhibition (9, 10, 11). These data reveal that the autoinhibited conformation is more complex than initially thought, with the distal region of the stalk (CC3 and CC4) and the unstructured C-terminal tail folding back onto the proximal stalk (CC1 and CC2), while the motor domains establish numerous intramolecular interactions. The release of this autoinhibited conformation upon adaptor binding can lead to motor activation (12, 13). KLC also contributes to kinesin-1 autoinhibition independently of KHC intramolecular interactions, by preventing kinesin–microtubule binding (14, 15, 16). These recent structural insights provide a compelling framework for understanding how adaptor proteins release the autoinhibited kinesin-1 and activate the motor.

**Figure 1:**
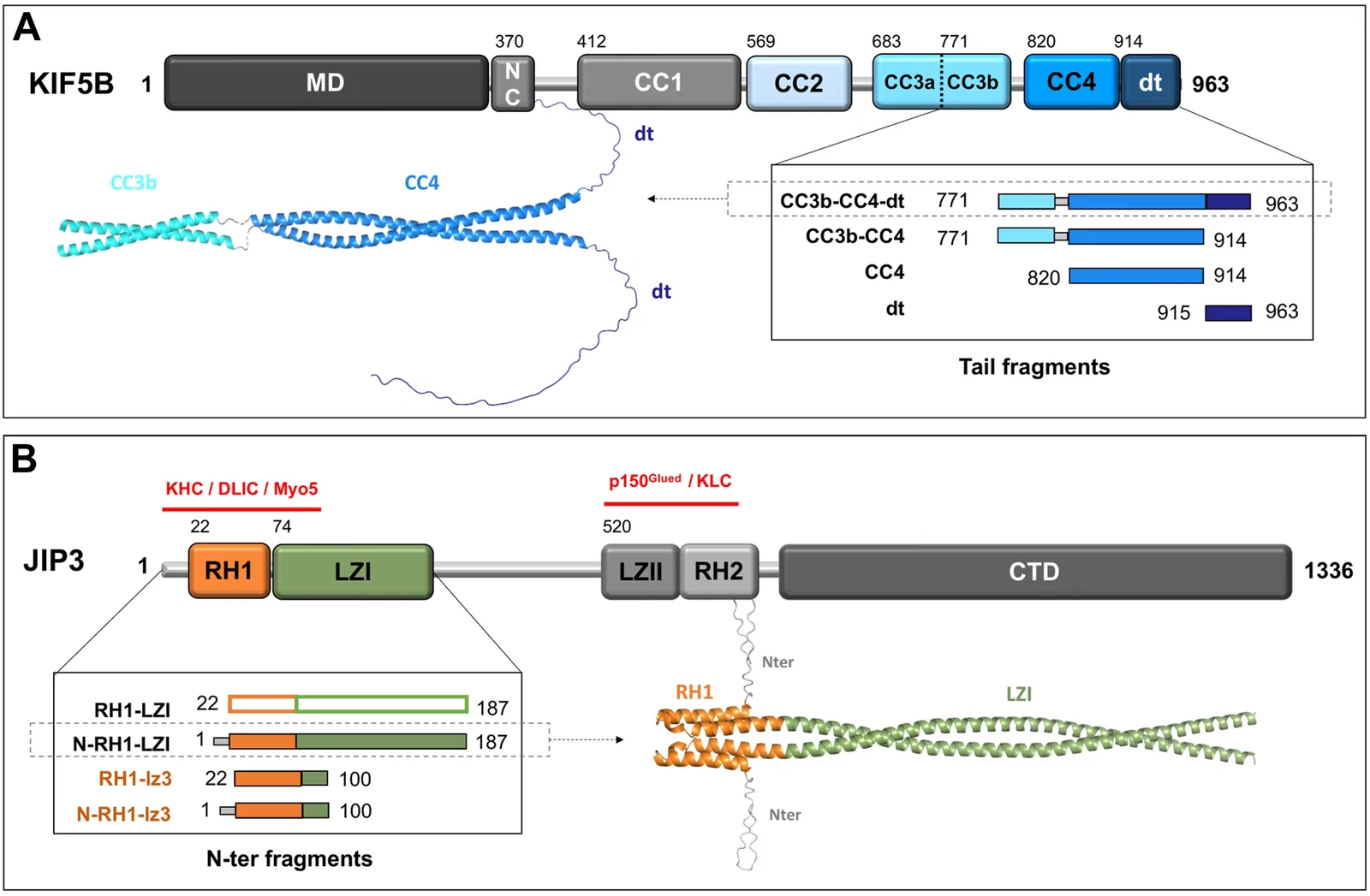
**The Tail region of KIF5B and the N-terminal part of JIP3.***A*, schema of the full length KIF5B. MD: Motor Domain; NC: Neck Coil; CC: coiled-coil; dt: distal tail. The *black-lined box* (*bottom*, *right*) shows the fragments of the Tail region of KIF5B used in this study. An AF2 model of the CC3b-CC4-dt fragment (*bottom*, *left*) is shown in cartoon. *B*, schema of the full-length JIP3 proteins. RH1 and RH2: RILP-Homology 1 and 2; LZI and LZII: Leucine Zipper I and II; CTD: C-Terminal Domain. Selected JIP3 partners are indicated in red. The *black-lined box* (*bottom*, *left*) shows the fragments of the N-terminal part of JIP3 used in this study. The AF2 model of the N-RH1-LZI fragment (*bottom*, *right*) is shown in cartoon. Both AF2 models are colored using the schema domain coloring.

JIP3 and JIP4 are motor–cargo adaptors involved in vesicle transport through their interactions with kinesin-1 (1, 6, 17) and the dynein–dynactin complex (2, 4, 18). They were initially identified as scaffold proteins that bind c-Jun N-terminal kinase (JNK), linking JNK signaling to vesicle transport (19, 20, 21). JIP3 is predominantly expressed in the nervous system (19, 21, 22), and JIP3-null mutant mice die shortly after birth (23, 24). JIP4, while also highly expressed in the brain, has a broader tissue distribution (20). JIP3 and JIP4 share several functional redundancies: (*i*) they are essential for postnatal brain development in mice (25); (*ii*) they are involved in neurite outgrowth and axonal elongation (5, 6, 26, 27); (*iii*) JIP3 and/or JIP4 knockout leads to lysosome mislocalization and accumulation, highlighting their role in axonal lysosome transport (18, 28, 29, 30), as well as autophagosome trafficking (31, 32). In invertebrates, there is only a single JIP3/4 homolog, unc-16 in *Caenorhabditis elegans* and Syd in *D*. *melanogaster*. Mutations in these genes lead to mis-sorting and mislocalization of Golgi and post-Golgi membranous axonal cargos (1, 21, 33, 34). JIP3 has been shown to interact with TrkB and participate in its anterograde transport to axon terminals, where it mediates BDNF-induced signaling (18, 35, 36). JIP4, on the other hand, is implicated in the regulation of endosomal tubulation during exocytosis (37), in recycling endosome delivery to the midbody during cytokinesis (38), and in endosome-to-trans-Golgi network cargo transport (39). Activation of kinesin-1 by JIP3 occurs through relief of its autoinhibited conformation (6). The direct interaction of JIP3 with KHC, independent of KLC, is functional and enhances kinesin-1 motility along microtubules (5, 6).

JIP3 and JIP4, which are closely related paralogs, consist of three folded domains: the RH1-LZI (RILP Homology 1-Leucine Zipper I) domain at the N-terminus, the LZII-RH2 (Leucine Zipper II- RILP Homology 2) domain in the central region, and a WD40 repeat domain at the C-terminus (Fig. 1*B*). The RH1-LZI and LZII-RH2 domains are separated by an extended intrinsically disordered region. These adaptors adopt a dimeric conformation through two coiled-coil regions (LZI and LZII). JIP3/4 recruit kinesin-1 *via* a dual binding mode, with the LZII domain binding KLC (40, 41), and the RH1-LZI domain binding the tail region of KHC (5). Using an *in vitro* TIRF-based motility assay, Watt *et al*. determined that one KHC dimer binds up to two JIP3 dimers (6). We previously characterized the RH1-LZI domain of JIP3/4 and demonstrated that it forms a stable dimeric structure approximately 210 Å in length, and that the RH1 domain is found exclusively in the RILP family (RILP, RILPL1, and RILPL2) and in JIP3/4 (42). The RH1 domain is involved in motor recruitment for both actin- and microtubule-based transport. For example, RILPL2 recruits myosin 5A (43, 44), while JIP3/4, RILP, and RILPL1 recruit the dynein light intermediate chain (DLIC) (2, 3, 45, 46, 47). Notably, only JIP3/4 is known to recruit KHC (5). The interaction of the RH1 domain of JIP3 with the dynein–dynactin complex, as well as that of RILPL2 with myosin 5A, has been structurally characterized (7, 43). However, the molecular details of the interaction between the RH1 domain of JIP3/4 and KHC remain unknown. Such structural information is crucial to understanding how this interaction contributes to kinesin-1 activation. In this study, we investigate the molecular basis of the interaction between JIP3/JIP4 and KIF5B.

We used a MicroScale Thermophoresis (MST) approach to determine the binding affinities between various fragments, chimera, and mutants of KIF5B and JIP3/4. This analysis allowed us to identify the minimal binding regions in both KIF5B and JIP3/4 that are required for their interaction, as well as mapped the KIF5B-binding surface on JIP3. Strikingly, our data revealed that KIF5B exhibits different binding affinities for JIP3 and JIP4, suggesting variability between these paralogs in kinesin-1 regulation. Furthermore, we extended our structural analysis to other kinesin-1 partners that bind to the CC4 region, highlighting a recurrent coiled coil–coiled coil mode of interaction. Finally, in light of recent models of kinesin-1 in its autoinhibited state, our findings offer insights into how the N-terminal region of JIP3/4, by binding to the CC4 segment of kinesin-1, could compete with the intramolecular interactions that maintain the auto-inhibited conformation and consequently relieve it.

## Results/discussion

### Characterization of KIF5B-Tail and JIP3/4-Nter fragments

In order to characterize KIF5B binding on JIP3/4 proteins, we designed various fragments of the Tail region of KIF5B (KIF5B-Tail) and the N-terminal part of JIP3 and JIP4 (JIP3/4-Nter), and validated their structural integrity using, Circular Dichroism (CD) and Multi-Angle Light Scattering (MALS), respectively (Fig.S1 and Tables S1 and S2). Collectively, our results showed that all KIF5B-Tail and JIP3/4-Nter fragments used here, exhibit the expected structural integrity require to perform binding assays. The Tail region of KIF5B consists of the CC3 coiled-coil encompassing the KLC-binding site, followed by the CC4 coiled-coil and the 50-aa unstructured distal tail (dt) (Fig. 1*A*). Based on combined secondary structure (Fig. S2) prediction analysis, we conceived four fragments of the Tail region of the human KIF5B (Fig. 1*A* and Table S1): (*i*) the last part of the CC3 coiled-coil (called here after CC3b) followed by the CC4 coiled-coil and the distal tail (CC3b-CC4-dt fragment), (*ii*) a truncated form lacking the distal tail (CC3b-CC4 fragment), (*iii*) the CC4 coiled-coil alone (CC4 fragment), and (*iv*) the distal tail alone (dt fragment). The CD characterization of the KIF5B-Tail fragments showed that the CC3b-CC4 and CC4 fragments are mainly alpha-helical with 78% and 77% helix content, respectively (Fig.S1*A* and Table S1). For the CC3b-CC4-dt fragment, the helix content decreases to 52%, with a turn/random content at 38% (Table S1). These differences with the two shorter fragments agree with the presence of the predicted unstructured distal tail. Altogether, helix content percentages observed for each fragment are consistent with those based on consensus secondary structure prediction (Fig. S2). We used the ratio of the molar ellipticities at 222 nm and 208 nm ([θ]222/[θ]208) to evaluate the presence of coiled-coil helices. For a non-interacting α-helix, the ratio has been shown to be 0.83, while for two-stranded coiled-coils, the ratio was calculated to be 1.03 (48). For the CC3b-CC4-dt and CC3b-CC4 fragments, the [θ]222/[θ]208 ratio is very close to 1 and for the CC4 fragment alone, it is equal to 1.02. Then, the CC3b-CC4 region in these fragments is well forming a coiled coil helix and the distal tail is unstructured, consistent with the AF2 model of the CC3b-CC4-dt fragment (Fig. 1*A*).

The N-terminal part of dimeric JIP3/4 encompasses an RH1 domain which assembles as a dimer of two anti-parallel helices (α1 and α2) forming a four-helix bundle (Fig. 1*B*). The RH1 domain is flanked by a short unstructured region in its N-terminus (N) and is followed by a Leucine Zipper (LZI) domain of 14 heptad repeats assembled in a coiled-coil (Fig. 1*B*) (42). For both JIP3 and JIP4, we used a previously characterized fragment that encompasses the RH1 domain followed by the entire LZI coiled-coil (RH1-LZI fragment, Fig. 1*B*) (42), as well as three new fragments (Fig. 1*B* and Table S2). The first one contains the N-terminus region (N-RH1-LZI fragment) and the two others were truncated after the third heptad repeat of LZI coiled-coil with or without the N-terminus (N-RH1-lz3 and RH1-lz3 fragments, respectively). SEC-MALS characterization of the JIP3/4 fragments showed that the JIP3-N-RH1-LZI fragment eluted in a single peak containing a species with a molecular mass of 42.7 ± 0.4 kDa (Fig.S1*B* and Table S2), indicating a dimeric form in solution, as for the JIP3-RH1-LZI fragment (42). Similarly, shorter N-RH1-lz3 and RH1-lz3 fragments of both JIP3 and JIP4 proteins are also dimers (Fig.S1*B* and Table 2). Thus, JIP3/4 are dimeric even without the N-terminus or the eleven C-terminal heptad repeats of the LZI, meaning that these regions are dispensable for dimerization. Previously, we showed that the RH1 domain alone is not stable in the absence of the full LZI coiled-coil (42). These results indicate that the first three heptad repeats of LZI are sufficient to stabilize the dimeric form of JIP3 and JIP4.

**Table 1:** KIF5B interaction with JIP3 fragments by MST

KIF5B	JIP3	Kd (nM)	S/N	Affinity (Factor-fold)	
				Decrease	Increase
CC3b-CC4-dt	N-RH1-LZI	18.8 ± 4	19	1	1
	RH1-LZI	50.5 ± 12.9	14.4	2.7	-
	N-RH1-lz3	14.7 ± 1.8	34.4	-	1.3
	RH1-lz3	79.3 ± 15.9	17	4.2	-
CC3b-CC4-dt	N-RH1-lz3	18.3 ± 2.2	34.1	1	1
CC3b-CC4		77.7 ± 12	22	4.2	-
CC4		113 ± 17.1	22.3	6.2	-
dt		-		-	-

The JIP3 fragments do not have a tag, whereas the KIF5B fragments have an N-terminal 6-histidine tag. JIP3 fragments are labelled with the dye NHS-NT-488 from Nanotemper. S/N: signal to noise.

**Table 2:** KIF5B-CC3b-CC4 interaction with JIP3 mutants by MST

JIP3-N-RH1-lz3 fragment^a^	Kd (nM)	S/N	Affinity (Factor-fold)	
			Decrease	Increase
JIP3-WT	148 ± 21.4	25.5	1	1
Negatively charged residues				
JIP3-E27A	215 ± 57.3	15	1.4	-
JIP3-E39A	394.6 ± 137	12.1	2.7	-
JIP3-E41A	204.4 ± 57.8	13.2	1.4	-
JIP3-E54A	225.9 ± 26.4	32.8	1.5	-
Positively charged residues				
JIP3-R38A	151.4 ± 27.8	20.2	1.0	1.0
JIP3-R42A	90.8 ± 12.6	26.1	-	1.6
Polar residue				
JIP3-N65A	117.9 ± 23	18.7	-	1.2

The 6-histidine tag was removed from the CC3b-CC4 fragment.

a The JIP3 WT and mutants are labelled with NHS-fluorescein from Thermo Fisher Scientific. There is a difference in Kd for the interaction of CC3b-CC4 and JIP3-N-RH1-lz3 from Table 1 that can be explained by the presence or absence of the Histidine tag in CC3b-CC4 and a different fluorophore for JIP3 and mutants. S/N; signal to noise.

### Variation in the N-terminus of JIP3 and JIP4

**Figure 2:**
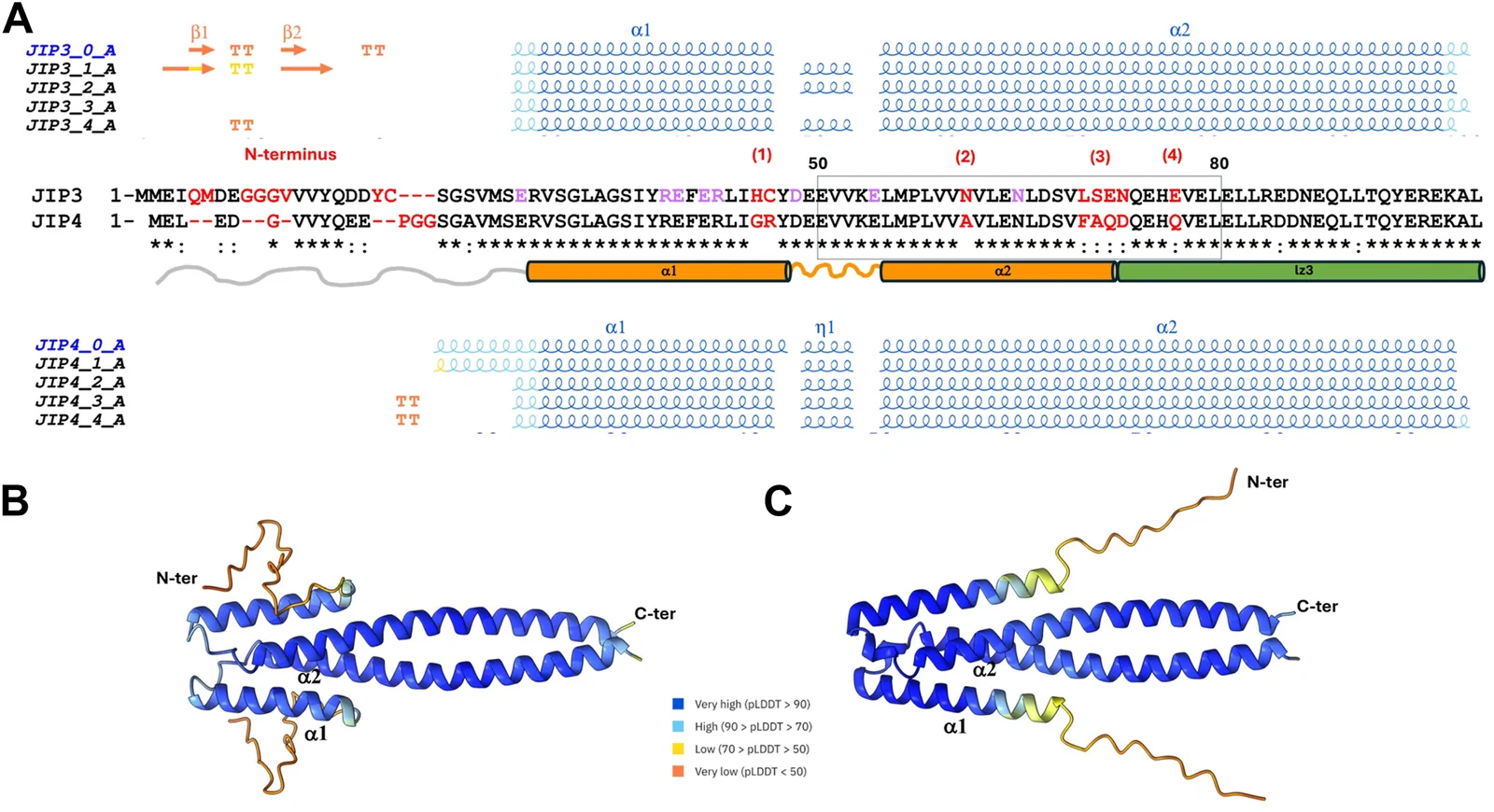
**Differences between JIP3 and JIP4 paralogs.***A*, the central part of the figure shows the alignment of the N-RH1-lz3 region of JIP3 and JIP4. Sequence differences are highlighted in *red*: the N-terminus and positions (1), (2), (3) and (4). The residues mutated in alanine to map the KHC binding site on the RH1 domain are colored in *violet* (Glu27, Arg38, Glu39, Glu41, Arg42, Asp48, Glu54, Asn61 and Asn65). A schematic representation of the secondary structure prediction from the analysis with Foldscript of the five models generated with AlphaFold-Multimer is shown in the *top* (JIP3) and the *bottom* (JIP4) (*B*) Cartoon representation of the highest ranked model generated for JIP3 (ranked_0 with pLDDT = 80.6; ipTM = 0.718 and pTM = 0.726) coloured by the pLDDT confidence measure. *C*, cartoon representation of the highest ranked model generated for JIP4 (ranked_0 with pLDDT = 86.3; ipTM = 0.753 and pTM = 0.756) coloured by the pLDDT confidence measure.

Previous studies on JIP3 and JIP4 were conducted using constructs lacking their unstructured N-terminal regions. However, these regions exhibit sequence variations between the two paralogs, which could influence protein recognition and/or interaction. The N-terminus of JIP4 is four residues shorter than that of JIP3 (Fig. 2*A*). Both regions share similar isoelectric points (3.45 and 3.33, respectively) and slightly different aliphatic indices (67.82 and 59.62, respectively). Notably, JIP4 has a substantially higher instability index (67.61) compared to JIP3 (46.82), as well as distinct GRAVY (Grand Average of Hydropathy) values (-0.145 and 0.012, respectively). Consequently, the N-terminus of JIP4 is predominantly hydrophilic, whereas that of JIP3 is more hydrophobic.

Using AlphaFold2-Multimer (49, 50, 51), we generated five models for the dimeric form of the N-RH1-lz3 fragment of JIP3 and JIP4 (Fig. 2*B*). Comparison of the five models using FoldScript (52) revealed that the N-terminus of JIP3 exhibits variability across the models: it may be disordered or have a propensity to fold into a β-sheet. In contrast, the N-terminus of JIP4 remains fully unstructured in all five models (Fig. 2*A*). The RH1-lz3 domain, however, is highly conserved across the five models. Superposition of these models yields RMSDs (Root-Mean-Square Deviations) ranging from 0.20 to 0.55 Å for JIP3 and from 0.29 to 0.59 Å for JIP4 (calculated on Cα atoms for 71 residues). The prediction of an unstructured N-terminus aligns with QELS (Quasi-Elastic Light Scattering) measurements of the JIP3 and JIP4 fragments. The N-RH1-lz3 fragments exhibit hydrodynamic radii (Rh) of 2.9 ± 0.2 nm and 2.7 ± 0.2 nm, respectively, while the RH1-lz3 fragments are smaller, with Rh values of 2.2 ± 0.2 nm for both JIP3 and JIP4 (Table S2). The differences of 0.7 nm and 0.5 nm are substantial for regions composed of only 21 residues (JIP3) and 17 residues (JIP4), respectively, and can be attributed to the presence of disordered regions.

### Mapping the binding regions for the interaction between JIP3 and KHC

To characterize the mode of binding of JIP3 to KIF5B, we used various fragments of both proteins and analyze their interaction in solution. Several biophysical techniques were tested, including AUC (Analytical Ultracentrifugation), BLI (Bio-Layer Interferometry), FIDA (Flow Induced Dispersion Analysis), MST (Microscale Thermophoresis), and ITC (Isothermal Titration Calorimetry), but only MST allowed us to monitor the interaction effectively. The dimeric coiled-coil domains of both proteins tended to form higher-order oligomeric states at high concentrations, complicating measurements by calorimetry due to elevated dilution heats and by ultracentrifugation due to the presence of multiple species. Additionally, these proteins exhibited significant non-specific interactions with sensors (BLI) or capillaries (FIDA), particularly KHC, likely due to its high isoelectric point (pI 10.24). We therefore analyzed all interactions using MST with coated capillaries and optimized buffers, which minimized non-specific interactions. The binding affinities determined here using a single technique are considered as relative measurements and thus are relevant for comparing Kd values between different fragments or between wild-type and mutant proteins.

**Figure 3:**
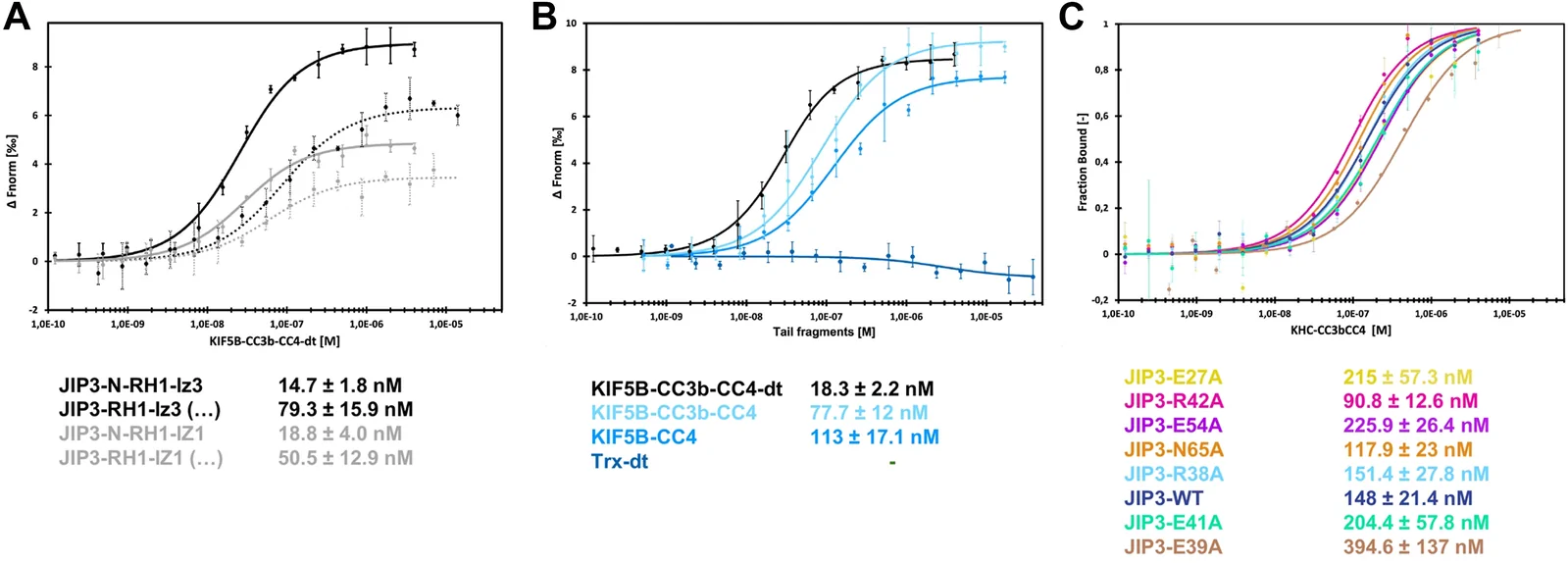
**Mapping the interaction of JIP3-Nter and KIF5B-Tail.***A*, MST analysis of Kif5b-CC3b-CC4-dt binding to different labeled fragments of JIP3: N-RH1-LZ1 (*gray line*), RH1-LZ1 (*gray dots*), N-RH1-lz3 (*black line*) and RH1-lz3 (*black dots*). *B*, MST analysis of KIF5B-Tail fragments (CC3b-CC4-dt (*black*), CC3b-CC4 (cyan), CC4 (*light blue*), and Trx-dt (*dark blue*) binding to labelled JIP3-N-RH1-lz3. *C*, Fluorescence analysis of KIF5B-CC3b-CC4 binding to labelled JIP3-N-RH1-lz3 alanine mutants. The concentration of the labelled JIP3-N-RH1-lz3 fragments and mutants was kept constant at 20 nm while the concentration of KIF5B fragments was varied. In this case, the change in fluorescence was used to determine the binding affinities, as we observed specific changes in the overall fluorescence intensity due to ligand binding.

First, we confirmed the interaction between KIF5B and JIP3 using the longest fragments of both proteins: KIF5B-Tail and JIP3-Nter. Specifically, the KIF5B-CC3b-CC4-dt fragment binds to the JIP3-N-RH1-LZI fragment with a dissociation constant (Kd) of 18.8 ± 4 nm (Fig. 3*A*, Table 1). We then tested shorter fragments of JIP3. When the LZI motif is truncated after the third heptad repeat (N-RH1-lz3 fragment), the Kd remains very similar compared to the N-RH1-LZI fragment. However, removal of the N-terminus (RH1-LZI fragment) reduces the binding affinity by 2.7-fold relative to the N-RH1-LZI fragment. A 4.2-fold reduction in affinity is observed when the N-terminus is removed from the shorter fragment (Fig. 3*A*, Table 1). Since the N-RH1-lz3 fragment interacts with KIF5B with almost the same Kd as the N-RH1-LZI fragment, we selected the shorter fragment for further characterization of the interaction with KHC.

To define the minimal region of KIF5B required for JIP3 binding, we analyzed and compared the interaction between the N-RH1-lz3 fragment of JIP3 and various truncated fragments of KIF5B-Tail. Removal of the distal tail in KIF5B resulted in a 4.2-fold decrease in binding affinity (CC3b-CC4-dt vs. CC3b-CC4; Fig. 3*B*, Table 1) with JIP3. However, no significant interaction was detected between JIP3 and the distal tail alone at the tested concentrations (in the absence of the CC3b-CC4 region; Fig. 3*B*, Table 1). These results suggest that the distal tail may stabilize the CC3b-CC4 coiled-coil or the JIP3-KIF5B complex. When the CC3b is removed, the CC4 region has a 1.5-fold decrease binding affinity for JIP3 (CC3b-CC4 vs. CC4; Fig. 3*B*, Table 1), indicating a small contribution of the CC3b region that can be due to the stabilization of the CC4 coiled-coil or the JIP3-KIF5B complex but a direct implication cannot be excluded. The binding affinity of the CC4 alone for JIP3 is still in the nanomolar range so we consider it as the minimal binding region for JIP3, but the flanking domains (CC3b and dt) have some influence on the interaction.

We have thus identified the CC4 region of KIF5B and the RH1-lz3 domain of JIP3 as the minimal interacting regions, and we have demonstrated that two unstructured regions: the N-terminus of JIP3 and the distal tail of KIF5B, modulate this interaction. These findings are consistent with previous studies that defined a minimal KIF5-binding site in JIP3 spanning residues 50 to 80 (5), which corresponds to the C-terminal part of the RH1 domain and the beginning of the LZI motif (Fig. 2*A*). Using GST pulldown titration experiments, Sun *et al*. reported a Kd of 1.83 ± 0.43 μm between the [1–772] region of JIP3 and the tail region of mouse KIF5C (residues 807–956, encompassing CC4 and the distal tail) (5). Notably, the CC4 region of mouse KIF5C and human KIF5B share 97.6% sequence identity (100% similarity), rendering them virtually identical. The discrepancy between the binding affinity reported by Sun *et al*. and the higher affinity we determined can be attributed to several factors: the methodology used (GST pull-down vs MST), the presence of a tag at the N-terminus of JIP3 (GST-tag vs no tag), and the size of the fragment analyzed (residues 1–772 vs 1–100). The interaction between JIP3 and KIF5B is expected to have a significant electrostatic component, as JIP3-Nter and KIF5B-Tail contain a high proportion of negatively charged residues (Glu and Asp; pI 4.2) and positively charged residues (Arg and Lys; pI 10.2), respectively. To further define the binding site within the RH1 domain that interacts with the CC4 of KIF5B, we identified solvent-exposed charged residues within the RH1 domain. We individually mutated the following residues to alanine: Glu27, Glu39, Glu41, Glu54, Arg38, Arg42, and Asp48 (Fig. 2*A*). We also mutated the polar Asn65 residue which is accessible for binding (Fig. 2*A*). The interaction between these mutants and KIF5B-CC3b-CC4 was then analyzed using MST (Fig. 3*C*, Table 2). The mutation of Asp48 to alanine somehow affects the protein, resulting in a loss of expression in bacteria, and thus could not be tested. All mutants retained the ability to interact with KIF5B, so none of these residues represent a hotspot for the interaction. However, mutations at Glu27, Glu39, Glu41, and Glu54 resulted in a modest decrease in binding affinity (1.4- to 2.7-fold). In contrast, mutations at Arg42 and Asn65 slightly increase binding affinity (1.6- and 1.2-fold, respectively), the mutation on Arg38 has no effect (Fig. 3*C*, Table 2). The removal of negatively charged residues in JIP3 results in a modest reduction of the affinity, this could indicate an extended interface of electrostatic interactions in which a single change in alanine is not sufficient to disrupt the interaction between JIP3 and KIF5B.

A similar situation, in which single mutations are not sufficient to modify the binding affinity of the complex between the CC4 domain and the atypical tropomyosin with its extended interface, was also observed (53). This kind of interface, extended and shallow, is common in coiled-coil interactions, for example in SNARE complexes (54), the autoinhibited conformation of kinesin-1 (10, 11), and the tetramer assembly of kinesin-5 (55). A single mutation in an extended interface can be more easily compensated by the cooperativity of the other residues, as well as by the potential for the coiled-coils to adopt a large number of alternative conformations.

### Difference in KIF5B binding affinity between JIP3 and JIP4

**Figure 4:**
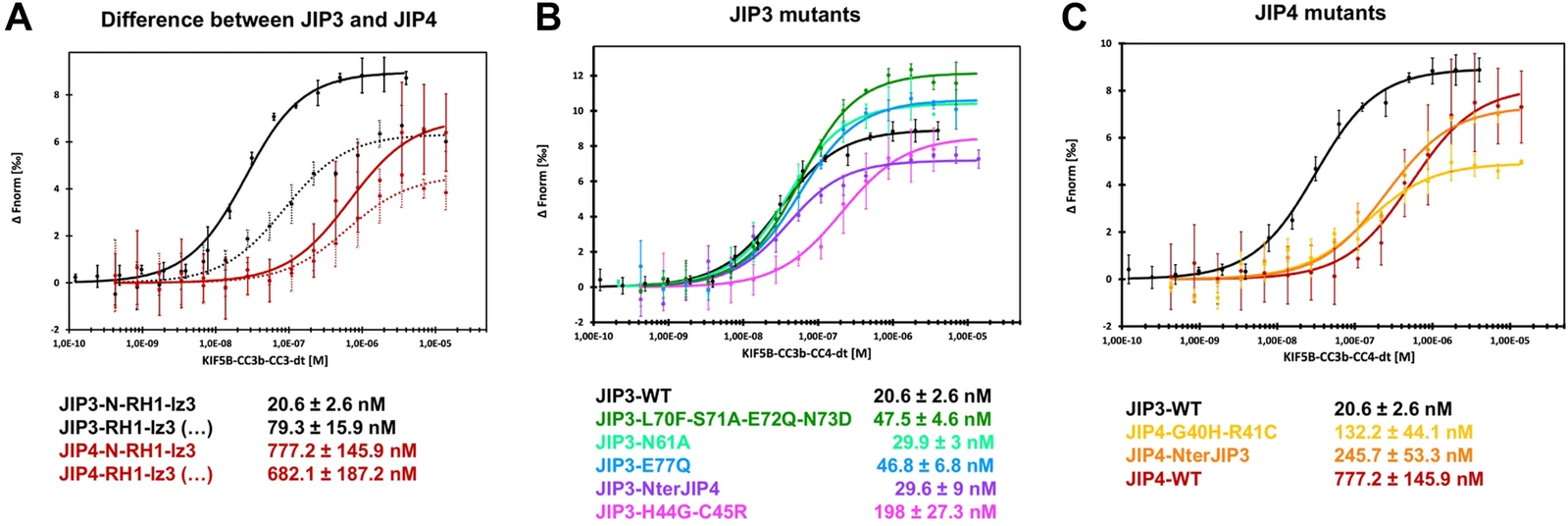
**KIF5B-Tail binds with different affinities to JIP3 and JIP4.***A*, MST analysis of the binding of KIF5B-CC3b-CC4-dt to labelled JIP3 and JIP4 fragments: JIP3-N-RH1-lz3 (*black line*), JIP3-RH1-lz3 (*black dots*), JIP4-N-RH1-lz3 (*red line*) and JIP4-RH1-lz3 (*red dots*). JIP4 binds with less affinity than JIP3. *B*, MST analysis of the binding of KIF5B-CC3b-CC4-dt to labelled JIP3 WT and mutants to JIP4 residues. *C*, MST analysis of the binding of KIF5B-CC3b-CC4-dt to labelled JIP4 mutants to JIP3 residues. For all the titrations, the concentration of the labelled JIP3 and JIP4 fragments and mutants was kept constant at 20 nm while the concentration of KIF5B-CC3b-CC4-dt was varied.

Strikingly, we observed that KIF5B exhibits a significantly weaker binding affinity for JIP4 than for JIP3. MST binding assays revealed that the KIF5B-CC3b-CC4-dt fragment binds to the JIP4-N-RH1-lz3 fragment with a Kd of 777.2 ± 145.9 nm, representing a 37.7-fold reduction in binding affinity compared to the equivalent JIP3 fragment. Moreover, unlike JIP3, the N-terminus of JIP4 does not influence KIF5B binding, as the JIP4-RH1-lz3 fragment binds to KIF5B with a similar Kd of 682.1 ± 187.2 nm (Fig. 4*A* and Table 3).

**Table 3:** KIF5B-CC3b-CC4-dt interaction with JIP4 by MST

JIP3/4	Kd (nm)	S/N	Affinity (Factor-fold)	
			Decrease	Increase
JIP3-N-RH1-lz3	20.6 ± 2.6	31.3	1	
JIP4-N-RH1-lz3	777.2 ± 145.9	20.3	37.7	1
JIP4-RH1-lz3	682.1 ± 187.2	13.7	-	1.1
JIP3-N^JIP4^-RH1-lz3	29.6 ± 9	12.4	1.4	-
JIP4-N^JIP3^-RH1-lz3	245.7 ± 53.3	16.9	-	3.2
JIP3^a^-N61A	29.9 ± 3	39.5	1.5	-
JIP3^a^-L70F-S71A-E72Q-N73D	47.5 ± 4.6	37.8	2.3	-
JIP3^a^-E77Q	46.8 ± 6.8	25.5	2.3	-
JIP3^a^-H45G-C46R	198 ± 27.3	29.3	9.6	-
JIP4^a^-G40H-R41C	132.2 ± 44.1	10.1	-	5.9

The Kd measurements are directly comparable, since all proteins were prepared under exactly the same conditions, and their characterization shows that they form dimers and no higher-order oligomers at the working concentrations.

a The N-RH1-lz3 fragment of JIP3 and JIP4 were used here. S/N; signal to noise.

To elucidate the basis for this difference in binding affinity, we identified sequence variations between the JIP3 and JIP4 paralogs (Fig. 2*A*) and investigated their role in KIF5B binding through JIP3/JIP4 sequence swapping (Table 3). Sequence alignment of JIP3 and JIP4 (Fig. 2*A*) revealed four key variations in the N-terminal region: (1) The His45-Cys46 residues at the end of the α1 helix in JIP3 are replaced by glycine and arginine in JIP4; (2) Asn61 in the α2 helix is substituted by alanine; (3) The Leu70-Ser71-Glu72-Asn73 stretch at the α2 helix–LZI junction is replaced by Phe-Ala-Gln-Asp; and (4) Glu77 in the LZI is replaced by glutamine. To probe the functional significance of these differences, we (*i*) designed two chimeric variants: a JIP4 variant incorporating the N-terminus of JIP3 (JIP4-N^JIP3^-RH1-lz3) and a JIP3 variant incorporating the N-terminus of JIP4 (JIP3-N^JIP4^-RH1-lz3) and (*ii*) generated mutants in JIP3, substituting each of the four sites with the corresponding JIP4 residues. The binding affinities of these chimeric variants and mutants for KIF5B were then measured using MST.

These results demonstrate that the N-terminus of JIP3 and JIP4 behaves differently in KIF5B binding. The affinity of the JIP3-N^JIP4^-RH1-lz3 chimera for KIF5B is 1.4-fold lower than that of the wild-type JIP3-N-RH1-lz3 (Fig. 4*B* and Table 3), whereas the JIP4-N^JIP3^-RH1-lz3 chimera exhibits a 3.2-fold increase in affinity compared to wild-type JIP4-N-RH1-lz3 (Fig. 4*C* and Table 3). This suggests that the N-terminus of JIP3 contributes to the interaction with KIF5B, while that of JIP4 does not. Regarding the RH1 domain mutants, no significant difference in KIF5B binding affinity was observed between wild-type JIP3 and the Asn61 mutant (Fig. 4*B* and Table 3). This is consistent with a recent study showing that mutation of the adjacent residue in JIP3, Val60 to glutamine (V60Q), does not disrupt interaction with mouse KIF5C (3). Together, these findings indicate that the 60 to 61 region of JIP3 is not involved in KIF5B binding. In contrast, both the E77Q and L70F-S71A-E72Q-N73D mutants exhibit 2.3-fold decreases in affinity, respectively, compared to wild-type (Fig. 4*B* and Table 3), suggesting a modest contribution of these sites to KIF5B binding. Most significantly, the H45G-C46R mutant displays a 9.6-fold reduction in affinity (Fig. 4*B* and Table 3), highlighting a role for these residues in KIF5B interaction. To validate this observation, we generated the equivalent JIP4 mutant, JIP4-N-RH1-lz3-G40H-R41C, which shows a 5.9-fold increase in affinity compared to wild-type JIP4 (Fig. 4*C* and Table 3). So, His45 and Cys46 in JIP3, which are solvent-exposed at the tip (α1-α2 loop) of the RH1 domain, are directly involved in the interaction (Fig. 2*A*). Notably, none of the individual mutants or chimeric fragments tested account for the 37.7-fold difference in binding affinity observed between JIP3 and JIP4. This suggests that the combined effects of the N-terminus and residues 45 to 46, and probably together with residues 77 and 70 to 73, are required to explain the disparity in KIF5B binding affinity between the two paralogs.

While the binding affinity of JIP3 for KIF5B is in the nanomolar range, that of JIP4 is significantly reduced. Despite this weaker affinity, comparable to the affinities measured for motor light chains (such as KLC (41) or DLIC (3)) bound to JIP3/4, JIP4 remains capable of recruiting KIF5B in a biologically significant manner. This is supported by functional studies demonstrating that JIP4 is recruited by the kinesin-1 heavy chain and participates in kinesin-based transport processes (25, 56). Consequently, we hypothesize that the observed difference in binding affinity between JIP3 and JIP4 underlies distinct mechanisms of KIF5B regulation, including motor activation.

### Overview of the JIP3/4:KIF5B interface

To elucidate the structural basis of JIP3/4:KIF5B interface, we investigated the 3D structure of the JIP3/4:KIF5B complex. Initially, we attempted to crystallize complexes using various fragments of JIP3 and KIF5B. Despite the high binding affinity measured between these proteins, no crystals of the JIP3:KIF5B complex were obtained in our experiments. We then turned to computational modelling using AlphaFold2 (49, 50, 51). Despite systematically varying the boundaries of the JIP3/4 and KIF5B sequences, the stoichiometry of the complex, the multiple sequence alignments, and the AlphaFold parameters (monomer or multimer mode), we failed to generate a reliable model. The challenges in predicting a structural model for the JIP3/4:KIF5B complex may come from the coiled-coil nature of both interacting proteins (57). Coiled-coils are highly versatile structural motifs (58) capable of adopting diverse tertiary and quaternary organizations upon partner binding. For example, the 3D structure of the actin-binding protein tropomyosin 1 (αTm1) bound to the CC4 region of *Drosophila* KHC revealed that the dimeric coiled-coil of αTm1 dissociates, with only a single helix associating with the KHC-CC4 dimeric coiled-coil to form a tripartite coiled-coil complex (53). Another type of structural rearrangement observed in coiled-coils involves changes in register, as seen in the dynein stalk domain and its activator BicD2 (43, 59). The inherent versatility of coiled-coil interactions, combined with the presence of disordered regions (*e*.*g*., the N-terminus of JIP3), complicates the prediction of such complexes.

**Figure 5:**
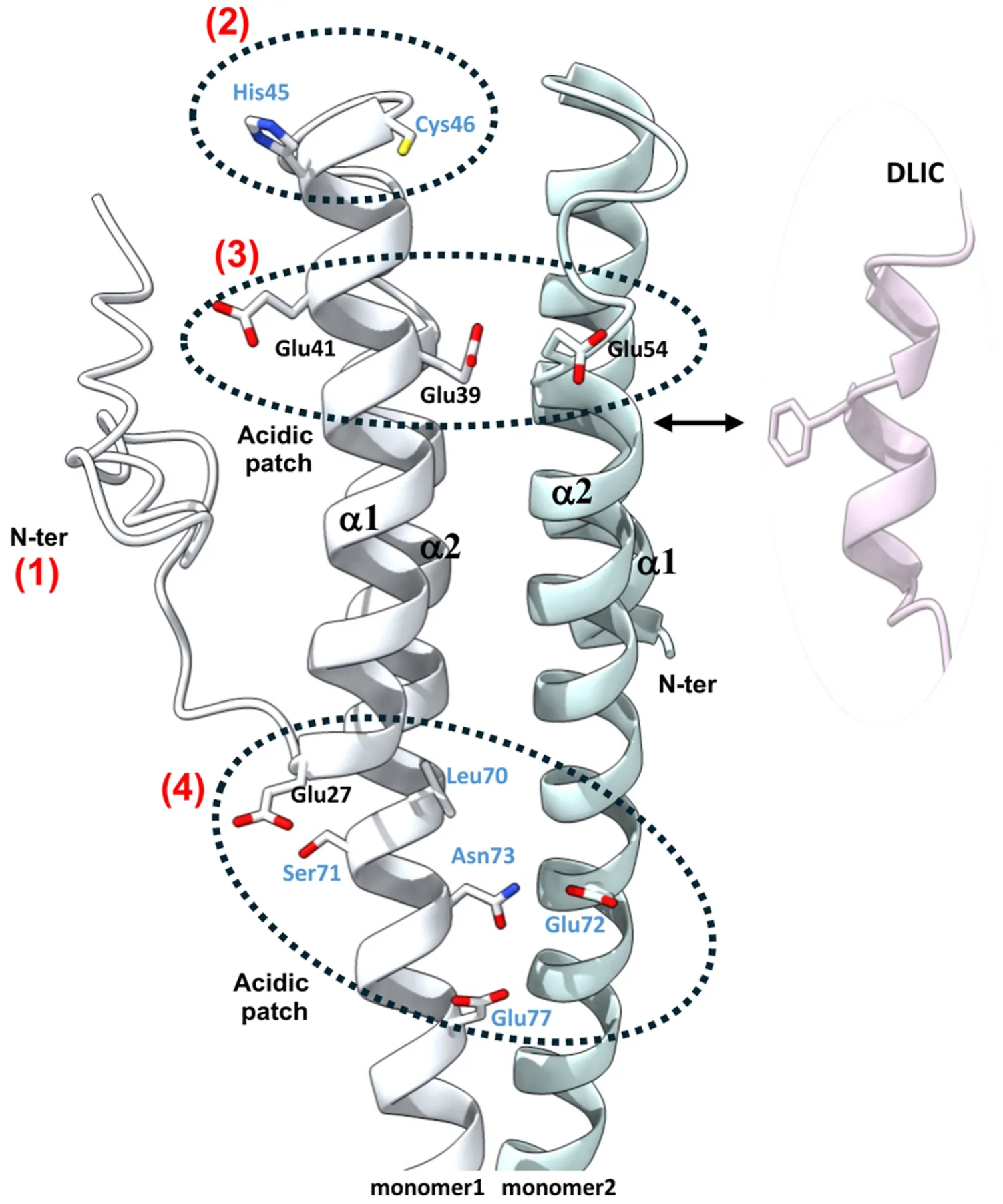
**JIP3 binds to the CC4 *via* four sites.** Cartoon representation of the dimer of the N-RH1-lz3 domain of JIP3. The disorder N-terminal domain of monomer2 is not shown for clarity. The residues having an influence in the interaction with KHC are shown in sticks, the *blue* labels indicate the residues different in JIP3 and JIP4. Four sites are implicated in the interaction: (1) the disordered N-terminus, (2) the His45-Cys46 residues at the tip of the RH1 domain, (3) an acidic patch composed of Glu39, Glu41, and Glu54 near the tip of the RH1 domain, (4) a second acidic patch, including Glu27, Glu72, and Glu77 near the junction of the RH1 and the LZI. A cartoon representation of a peptide of DLIC (*pink*) is shown to indicate its binding site: the hydrophobic pocket between a1 and a2 on the same monomer.

Taken together, our MST binding assays using various chimeras and mutants of JIP3/4-Nter enabled us to map the KIF5B-CC4 binding site. Specifically, we did not identify individual hotspot residues for the interaction of JIP3/4 with KIF5B. Instead, our findings suggest a multi-site interaction model, where each site moderately affects the interaction alone, but their collective effect is significant, such as the 37.7-fold difference in affinity observed between JIP3 and JIP4 that required the combined effect of distinct sites. We identified four sites for the interaction: (1) the disordered N-terminus, (2) the His45-Cys46 residues at the tip of the RH1 domain, (3) an acidic patch composed of Glu39, Glu41, and Glu54 near the tip of the RH1 domain, and (4) a second acidic patch, including Glu27, Glu72, and Glu77, located approximately 30 Å away from the first patch toward the LZI coiled-coil (Fig. 5*A*). Notably, these multiple sites define an extended KIF5B-binding surface, spanning from the tip of the RH1 domain to the beginning of the LZI. The role of the disordered N-terminus in this interaction remains unclear. Finally, in light of recent work, the human light intermediate chain (DLIC) binds to the RH1 domain of JIP3 and JIP4 by inserting the aromatic ring of a Phenylalanine into the hydrophobic pocket between helices α1 and α2. They also showed that this interaction does not overlap with KHC binding to JIP3 residues 1 to 108 (3, 7). The KIF5-binding surface we delineate here does not overlap with the proposed DLIC binding site, consistent with these previous reports (3, 7). Thus, the RH1 domain contains two distinct motor-binding zones: (*i*) a hydrophobic patch between helices α1 and α2 from the same monomer, where DLIC or MyoV interact *via* hydrophobic residue insertion (3, 7, 43), and (*ii*) an extended surface, predominantly acidic, between helices α1 and α2 from distinct monomers, which mediates interaction with KHC (Fig. 5*A*).

### The CC4 segment of KIF5 preferentially recruits coiled-coil partners

**Figure 6:**
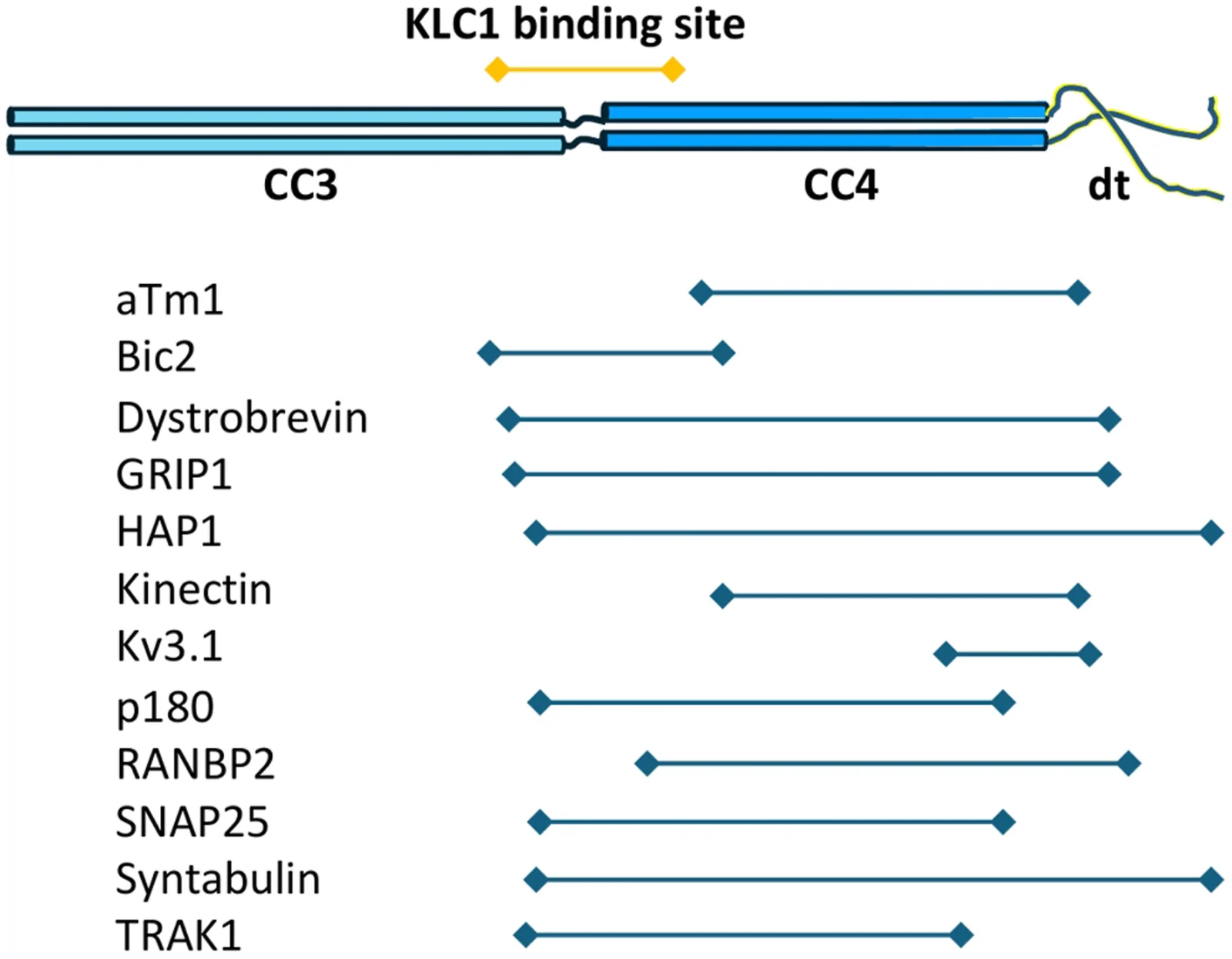
**The CC4 is a binding site for multiple proteins.** Schematic representation of the CC3-CC4-dt. Proteins interacting with the CC4 region or a part of it are listed. The *blue lines* indicate the interacting region corresponding to KIF5B. The KLC1 binding site, between the CC3 and CC4 is indicated with a yellow line.

The CC4 segment of KHC serves as a binding region for multiple proteins, including the JIP3/4 adaptors, SNARE proteins (SNAP25 and SNAP23), the ribosome receptor p180, the Milton/TRAK adaptor, the atypical tropomyosin αTm1, HAP1, RanBP2, GRIP1, syntabulin, dystrobrevin, Kv3.1, kinectin, and ankyrin G (53, 60, 61). We hypothesized that these binding partners share a conserved sequence or structural motif for interaction with KHC. To date, the only available 3D structure of KHC in complex with a partner is that of αTm1 bound to KHC-CC4 (53). For other partners, interacting regions or domains have been mapped using yeast two-hybrid assays, pull-down experiments, and immunoprecipitation; these methods provide only qualitative information (Table S3 and Fig. 6). To further investigate these interactions, we generated AlphaFold models of the partner binding regions to examine the folding of their KHC-interacting domains. While no common motif resembling the RH1 domain was identified, nearly all binding partners share key structural features: helical or coiled-coil conformations in the interacting regions, intrinsically disordered regions, and an acidic isoelectric point (Fig. S3). These characteristics appear critical for establishing interactions with CC4 and may reflect a general structural mechanism for CC4 recognition. Additionally, the 3D models of the autoinhibited form of kinesin-1 reveal extensive interactions between the CC1 and CC4 coiled-coils (10, 11). This observation further supports the notion that coiled-coil structures represent a favor motif for CC4 binding.

### Insights into how JIP3/4 relieve the autoinhibited form of KHC

**Figure 7:**
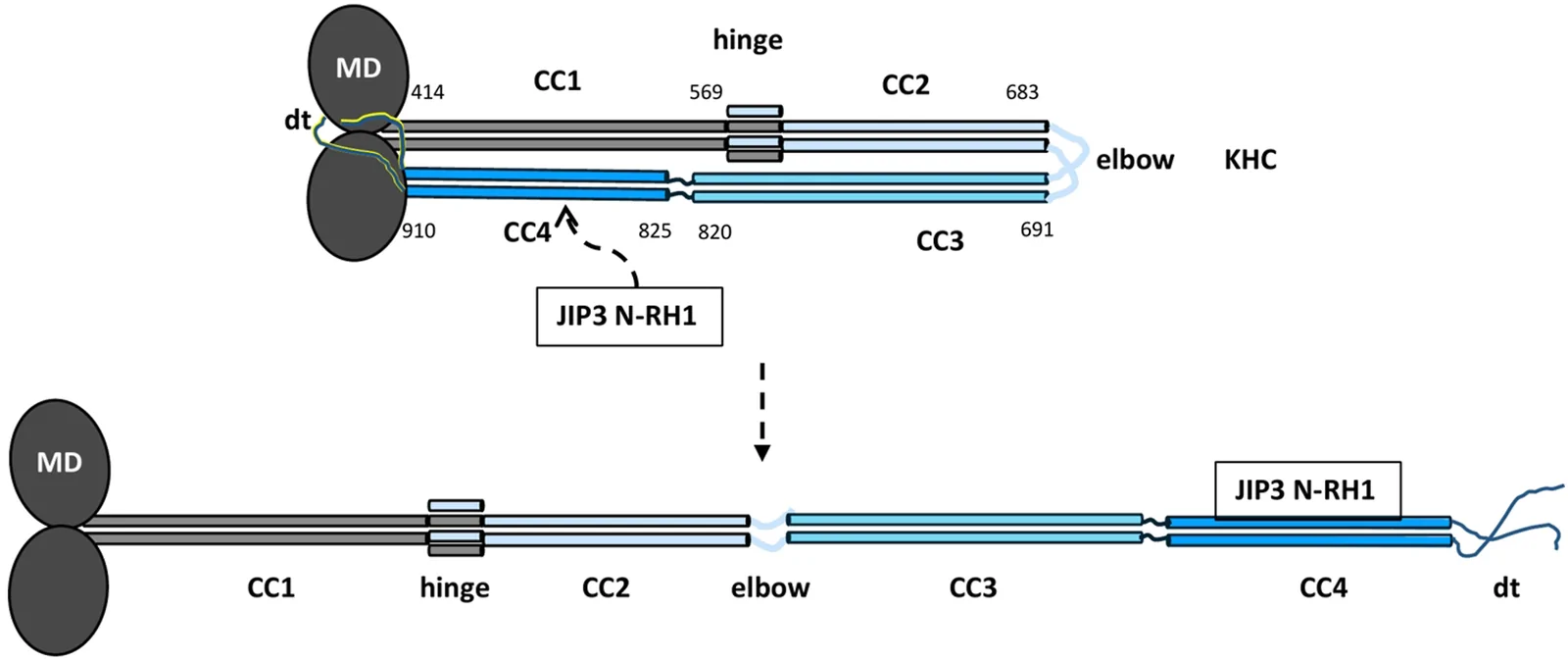
**Schematic representation of the activation of KHC by JIP3.** The auto-inhibited state of KHC is maintained by multiple interactions (grey doted lines) within the coiled coil regions, the motor domains and the non-structured tails (dt). The interaction of the N-RH1 region of JIP3 with the CC4 region of KHC is able to release the CC4 from its interactions with the CC1 and the motor domain to promote motility.

The full-length kinesin-1 autoinhibited conformation (with or without KLC) has been structurally characterized using single-particle negative-stain electron microscopy combined with AlphaFold2 predictive modelling and cross-linking mass spectrometry (10, 11). These studies demonstrate an autoinhibited conformation stabilized by multiple intramolecular interactions: the coiled-coil regions of the stalk interact with each other and with the motor domains (Fig. 7). Specifically, the CC4 segment of KHC, which constitutes the JIP3-binding site, forms extensive interactions along the CC1 coiled-coil and with the motor domains. Previous work has shown that the N-terminal region of JIP3 (residues 3–239) enhances KHC motility (5). We hypothesize that by binding with high affinity to the CC4 segment, JIP3/4 adaptors can directly compete with the CC1 and motor domains, thereby releasing the CC4 from these interactions to disrupt the autoinhibited conformation (Fig. 7). For example, in the presence of KLC, the autoinhibited conformation of kinesin-1 is maintained, and the intramolecular interactions are reinforced. The CC4 segment establishes additional contacts with both the N-terminal helical segment and the TPR domain of KLC. In this context, JIP3/4 can interact with both chains of kinesin-1: with KHC-CC4 *via* the N-RH1-lz3 domain and with KLC-TPR *via* the LZII domain. This could release the CC4 and TPR domains from their autoinhibiting intramolecular interactions and prime kinesin-1 activation. We cannot exclude that other regions of JIP3/4 may also interact with kinesin-1 or that other factors are needed for full activation. Recent studies show a more complex mechanism for KHC activation, in which multiple actors must act synergistically to fully activate kinesin-1 (16, 62).

## Conclusion

The characterization of JIP3 and JIP4 binding to KIF5B, as presented in this study, provides new insights into how KHC is recruited by these motor-cargo adaptors. Our data reveal that KIF5B-CC4 recruits the N-terminal region of JIP3/4, which includes both the disordered N-terminus and the RH1 domain. Within this region, no single residue was identified as a hotspot for the interaction with KIF5B; rather, we mapped an extended, predominantly acidic surface that is involved in KIF5B binding. This binding surface spans from the top of the RH1 domain to the beginning of the LZI, with the N-terminus modulating the interaction with KIF5B. Thus, the mode of binding of JIP3 with KHC, with a Kd in the nanomolar range, is not mediated by a single hotspot interaction, as observed between JIP3 and DLIC (Kd in the micromolar range; (3)). Instead, it involves multisite interactions, where the disordered N-terminus, the top of the RH1 domain, and the acidic patches located between the α1 and α2 helices from different monomers act synergistically. This complexity may explain the difficulty in obtaining a reliable model of the JIP3-KHC complex, which is consistent with the challenges typically associated with coiled-coil interactions.

## Experimental procedures

### Gene constructs, protein expression and purification

cDNAs encoding the tail region of human KIF5B (KIF5B; accession number NP_004512.1) was cloned into the pET28 plasmid between *NdeI/Xho1* restriction sites. The KIF5B-CC3-CC4-dt (residues 771–963) was a kind gift of Reza Khayat, (City College of New York, New York, USA). The CC3-CC4 and CC4 fragments of KIF5B (residues 771–914 and 820–914, respectively) were conceived by restriction enzyme cloning in the same plasmid. cDNAs encoding the distal tail (dt) region (residues 916–963) of human KIF5B was cloned into the pETG-20A plasmid between *KpnI/Xho1* restriction sites, as a Thioredoxin-His fusion protein (Trx-dt). The recombinant expression of all KIF5B fragments was performed in *Escherichia coli* Rosetta. Bacteria cells were collected after induction with 0.3 mm IPTG, O/N at 20°C. Bacteria were suspended in Tris HCl 50 mm pH 8.0 buffer, incremented with 500 mm NaCl, 25 mm Imidazole, 5% Glycerol, 0.5 mm EDTA, 1 mm DTT, with the addition of anti-proteases and benzonase. After cell disruption by sonication, the lysate was centrifuged at 22,000*g* for 30 min at 4 °C and the supernatant was loaded on a His-Trap 5 ml column (Cytiva) equilibrated with 50 mm Tris pH 8.0, 500 mm NaCl, 5% Glycerol, 1 mm DTT, 0.5 mm EDTA and 25 mm Imidazole. A wash step was performed with the same buffer for CC3-CC4, incremented with 50 mm Imidazole pH 8.0, for CC4 and Trx-dt, with 125 mm Imidazole pH 8.0 for CC3-CC4-dt, followed by an elution step with 500 mm Imidazole pH8.0. Then, KIF5B-tail fragments were purified by gel filtration on a HiLoad 16/60 Superdex 75 column (GE Healthcare) in 20 mm Tris pH 8.0, 200 mM NaCl, and stored at −80 °C. The SDS gel of the different KHC fragments is shown in Figure.S1*A*.

Gene construct, protein expression and purification and characterization of the RH1-LZI fragment of JIP3 are described in (42). cDNAs encoding the N-terminal part of mouse JIP3-N-RH1-LZ1 (residues 1–187; NP_038959.2) was cloned into the pnEAtHX (kind gift of Didier Busso, CEA, Saclay, France) plasmid between *NdeI/BamHI* restriction sites. cDNAs encoding the N-RH1-lz3 and RH1-lz3 fragments of mouse JIP3 (residues 1–100 and 22–100, respectively) were cloned into the pETG20A plasmid between *Kpn1/Xho1* restriction sites. The JIP3 to JIP4 mutants were done by site directed mutagenesis using the N-RH1-lz3 plasmid as source. All JIP3 fragments and mutants were produced in *E*. *coli* Rosetta as N-terminus His-thioredoxin tag fusion proteins. For the N-RH1-LZ1 fragment, cells were collected after induction with 0.5 mM IPTG for 6 h at 30°C; for the N-RH1-lz3 and RH1-lz3 fragments cells were collected after induction with 0.5 mM IPTG O/N at 20 °C. Bacteria were suspended in 50 mM Tris pH 8.0, 500 mM NaCl, 5% Glycerol, 1 mM DTT, 25 mM Imidazole, 1 mM EDTA and anti-proteases. After cell disruption by sonication, the lysate was centrifuged at 22,000*g* for 30 min at 4 °C and the supernatant was loaded on a His-Trap 5 ml column (GE Healthcare) equilibrated with 50 mm Tris pH 8.0, 500 mm NaCl, 5% Glycerol, 1 mM DTT, 0.5 mM EDTA and 25 mM Imidazole pH 8.0. Protein elution was performed in the same buffer containing 500 mM Imidazole. The eluted protein was subjected to gel filtration on a HiLoad 16/60 Superdex 75 column (GE Healthcare) in 20 mM Tris pH 8.0 and 200 mm NaCl, followed by an overnight incubation with thrombin in order to cut the His-thioredoxin tag. Finally, all cleaved fragments were purified by a gel filtration on a HiLoad 16/60 Superdex 75 column (GE Healthcare) in 20 mM Tris pH 8.0 and 200 mM NaCl. All JIP3 fragments were stored at −80 °C. The SDS gel of the different JIP3/4 fragments is shown in Figure.S1*B*.

### AlphaFold2 structure prediction

The prediction of JIP3, JIP4 and KIF5B homodimers were performed using AlphaFold 2.3 through a local installation of ColabFold 1.5.5 (49, 51) running the MMseqs2 homology search program (63) to generate a multiple sequence alignment (MSA) against the UniRef30 clustered database (version 2202). Standard parameters were used for the prediction (3 recycles, 5 generated models with a single seed). For the prediction of the JIP3:KIF5B complex, sequences of human KIF5B and JIP3 were retrieved from UniProt (Consortium *et al*., 2020) and used as input in AlphaFold2-multimer (49, 50, 51). We filtered the resulting MSAs to remove spurious homologs by discarding sequences with less than 20% sequence identity to JIP3 and sequences with less than 35% sequence identity and 60% coverage for KIF5B. Next, full-length sequences were retrieved and re-aligned with MAFFT (64). We used either paired MSAs (KIF5B and JIP3 MSAs concatenated by matching sequences of the same species), unpaired MSAs or paired + unpaired MSAs. The MSAs were used as inputs to generate 5 structural models for the KIF5B:JIP3 complex, using a local version of the ColabFold interface 1.3.0 (51) running 6 iterations (recycles) of AlphaFold-Multimer (49, 50), with 5 different random seeds, on an NVIDIA A100 80Go GPU card. Different delimitations of KIF5B and JIP3 were tested (KIF5B: CC3-CC4-dt [771–963], CC4-dt [820–963] and CC4 [820–914] and JIP3: N-RH1-LZ1 [1–150], N-RH1-lz3 [1–100] and RH1-lz3 [20–100]). Stoichiometries of 2:2, 2:1 and 1:1 for KIF5B:JIP3 were tested. Whatever the MSA mode (paired, paired + unpaired or unpaired), the delimitations and the stoichiometry, we were not able to identify an interaction between KIF5B and JIP3 in the predicted models, which all have high confidence for the KIF5B and JIP3 folds and homodimers, but low confidence in the KIF5B-JIP3 potential interaction zone.

### Microscale thermophoresis

JIP3-Nter fragments were labelled using the Protein Labelling Kit Blue-NHS (NanoTemper Technologies). The labelling reaction was performed according to the manufacturer’s instructions in the supplied labelling buffer, applying a concentration of 20 μm protein (molar dye: protein ratio ≈ 3:1) at room temperature for 30 min. The JIP3 mutants to alanine were labelled with NHS-fluorescein (Thermo Scientific) using the same protocol as for the Blue-NHS. The labelled proteins were stored in buffer containing 20 mm Tris-HCl pH 8 and 200 mm NaCl. For the binding assays, the labelled JIP3-Nter fragments were adjusted to 40 nm with an assay buffer containing 50 mm Tris pH 8.0, 200 mm NaCl, 10 mm MgCl2, 0.05% Tween 20. KIF5B fragments were buffer exchanged to the assay buffer, and a series of 16 1:1 dilution was prepared. For the measurement, each KIF5B fragment dilution was mixed with the same volume of labelled JIP3-Nter fragments and mutants, which led to a final concentration of JIP3-Nter of 20 nm and a final KIF5B fragment concentration ranging from 120 pM to 4 μm (up to 38.2 μm when needed). The samples were loaded into Monolith NT.115 Premium Capillaries MO-K025 (NanoTemper Technologies). MST was measured using a Monolith NT.115 instrument (NanoTemper Technologies) at room temperature. Instrument parameters were adjusted to 40% LED power and low MST power. Data of three independent replicates were analyzed with the MO. Affinity Analysis software version 2.3 (NanoTemper Technologies) using the Kd fit model that describes a molecular interaction with a 1:1 stoichiometry according to the law of mass action. In the case of JIP3/4 fragments, KIF5B fragments and JIP3 *versus* JIP4 mutants, the signal from an MST-on time at 0.5 to 1.5 s was used for the analysis. In the case of the JIP3-N-RH1-lz3 alanine mutants labelled with fluorescein, we observed ligand-binding-specific changes in the overall fluorescence intensity, so the change in fluorescence was used to determine the binding affinities.

## Data availability

All the raw data will be available upon request.

## Supporting information

This article contains supporting information (65).

## Conflict of interest

The authors declare that they have no conflicts of interest with the contents of this article.
