# Supplementary material for: Insights into how JIP3 and JIP4 adaptor proteins relieve the auto-inhibited form of the kinesin-1 motor
Source: J Biol Chem. 2026 Jul 22;302(9):113355. doi: 10.1016/j.jbc.2026.113355 (PMC13499124; doi:10.1016/j.jbc.2026.113355)
Supplement: Supplementary file 1 [file mmc1.pdf]

SUPPLEMENTARY INFORMATIONS

SUPPLEMENTARY MATERIELS AND METHODS

Size exclusion chromatography with multi-angle light scattering analysis - Protein samples were analyzed by SEC-MALS (Size-Exclusion Chromatography coupled with Multi-Angle Laser Light Scattering). All JIP3/4 fragments were loaded on a Superdex 200 10/300 GL increase column (Cytiva) equilibrated with 20 mM Hepes pH 7.5 and 200 mM NaCl using a flow rate of 0.5 mL/min on a HPLC system (Shimadzu). Static light scattering and refractive index (RI), as well as UV absorbance at 280 nm signals were measured with a MiniDAWN TREOS / Optilab refractive detector (Wyatt Technology), and UV detector SPD-M20A (Shimadzu), respectively. The data were analyzed using ASTRA® 6.1 software (Wyatt Technology).

Circular dichroism experiments - The circular dichroism (CD) spectra of KIF5-Tail fragments were recorded on a Jasco J-810 spectrometer (Japan Spectroscopic Co., Tokyo, Japan) at room temperature in 20 mM sodium phosphate pH 7.5 and 200 mM NaF for all proteins. All spectra were recorded using a 0.1-mm path length quartz cell. Each spectrum is an average of 6 scans recorded using a 50 nm/min continuous scanning in the range 260-185 nm. Secondary structure analysis was performed using CONTINLL and CDSSTR software, for KIF5 [1].

Reference

[1] S. Narasimha, W.W. Robert, Estimation of Protein Secondary Structure from Circular Dichroism Spectra: Comparison of CONTIN, SELCON, and CDSSTR Methods with an Expanded Reference Set, Anal. Biochem. 287 (2000) 252–260. https://doi.org/10.1006/abio.2000.4880.

SUPPLEMENTARY TABLES

Table S1. Biophysical characterization of the C-terminal part of KIF5B

Fragment			CD (%)			
**name**	**Limit**	**Mw*** (kDa)	**α-helix**	**β-sheet**	**Turn/ Rand.**	**[θ]222 / [θ]208 ratio**
**CC3b-CC4-dt**	[771-963]	23.7	55	6	38	0.95
**CC3b-CC4**	[771-914]	18.9	78	4	17	0.99
**CC4**	[820-914]	13.3	77	7	17	1.02
**dt**	[916-963]	19.4	N.D.			

N.D. Not Determined**;** Rand., Random; * Mw is calculated considering the Trx-His tag fusion for the KIF5B-dt fragment and the His-tag fusion for other KIF5B fragments which correspond to the molecule used in structural and biophysical experiments.

Table S2. Biophysical characterization of the N-terminal part of JIP3 and JIP4.

Fragment				MALS		
**name**		**Limit**	**Mw**** (kDa)	**Mw** (kDa)	**Rh** (nm)	**f/f0**
**JIP3**	**N-RH1-LZI**	[1-187]	22.6	42.7 ± 0.4	4.5 ± 0.3	1.9
	**RH1-LZI***	[22-187]	20.3	39.2 ± 0.4	4.1 ± 0.3	1.8
	**N-RH1-lz3**	[1-100]	11.8	19.6 ± 0.3	2.9 ± 0.2	1.5
	**RH1-lz3**	[22-100]	9.6	17.1 ± 0.2	2.2 ± 0.2	1.25
**JIP4**	**N-RH1-lz3**	[1-95]	11.2	20.1 ± 0.4	2.7 ± 0.2	1.5
	**RH1-lz3**	[17-95]	9.5	17.7 ± 1.2	2.2 ± 0.2	1.25

* Fragment previously characterized in (Vilela, 2019). ** Mw is calculated considering the remaining sequence of the protease site after cleavage for JIP3-Nter fragment, which correspond to the molecule used in structural and biophysical experiments.

Table S3. Proteins that interact with the CC4 of KHC

Protein	kinesin	Source	Binding region in kinesin-1	Method	Kinesin-1 activation	References
Bic2	KHC	*Drosophila*	792-836	TIRF	Relieves auto-inhibition	Ali et al, 2025
Dystrobrevin	KIF5A	Mouse	804-934	Pull down<br>SPR	ND	Ceccarani et al, 2005
GRIP1	KIF5A<br>KIF5B	Rat	807-934<br>808-963	Y2H	Not alone, in cooperation with HAP1	Setou, et al, 2002
HAP1	KIF5B	Mouse	814-963	Pull down	Relieves auto-inhibition, full activation in cooperation with GRIP1	Twelvetrees et al, 2010<br>Twelvetrees et al, 2019
kinectin	KIF5A	Human	836-890	Y2H	ND	Ong et al, 2000
Kv3.1	KIF5B	Mouse	865-934	Pull down assay	ND	Xu et al, 2010
p180	KIF5B	Human	814-907	Y2H,<br>*in vitro* pull down	ND	Diefenbach et al, 2004
RANBP2	KIF5B	Mouse	826-935	Y2H,<br>*in vitro* pull down	Relieves auto-inhibition	Cho et al, 2007<br>Cho et al, 2009
SNAP25	KIF5B	Human	814-907	Y2H,<br>*in vitro* pull down	ND	Diefenbach et al, 2002
Syntabulin	KIF5B	Human	814-963	Pull down	ND	Su et al, 2004
αTm1	KHC	*Drosophila*	833-915	Y2H,<br>Pull down<br>3D structure	Inhibition	Dimitrova-Paternoga et al, 2021<br>Gaber et al, 2025
Milton/<br>TRAK1	KHC<br>/KIF5B	*Drosophila*	810-891	Co-IP/Co-IP	Relieves auto-inhibition	Glater et al, 2006<br>Van Sprosen et al, 2013<br>Henrichs et al, 2020

KHC: kinesin heavy chain. ND: Not Determined. Y2H: Yeast Two Hybrid. Co-IP: Co-immunoprecipitation. SPR: Surface Plasmon Resonance

SUPPLEMENTARY FIGURES

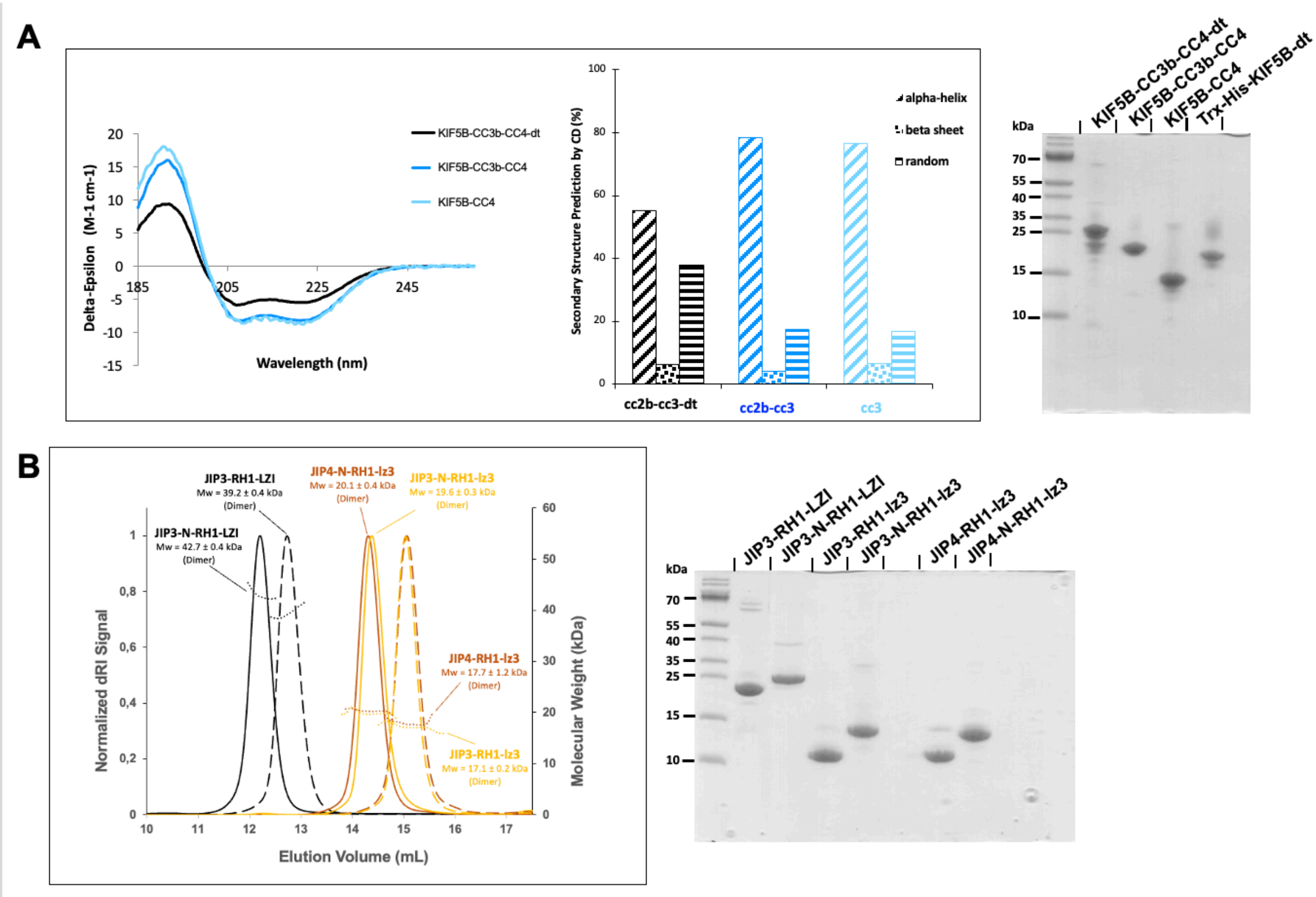

Figure S1. Biophysical characterization of the Tail region of KIF5B and the N-terminal part of JIP3/4. (A) CD spectra of the KIF5B-Tail fragments. On the right a SDS Gel of the different fragments used is shown. **(B)** SEC-MALS experiments on JIP3/4-Nter fragments. Superposition of MALS experiments on the JIP3 and JIP4 N-terminal fragments.

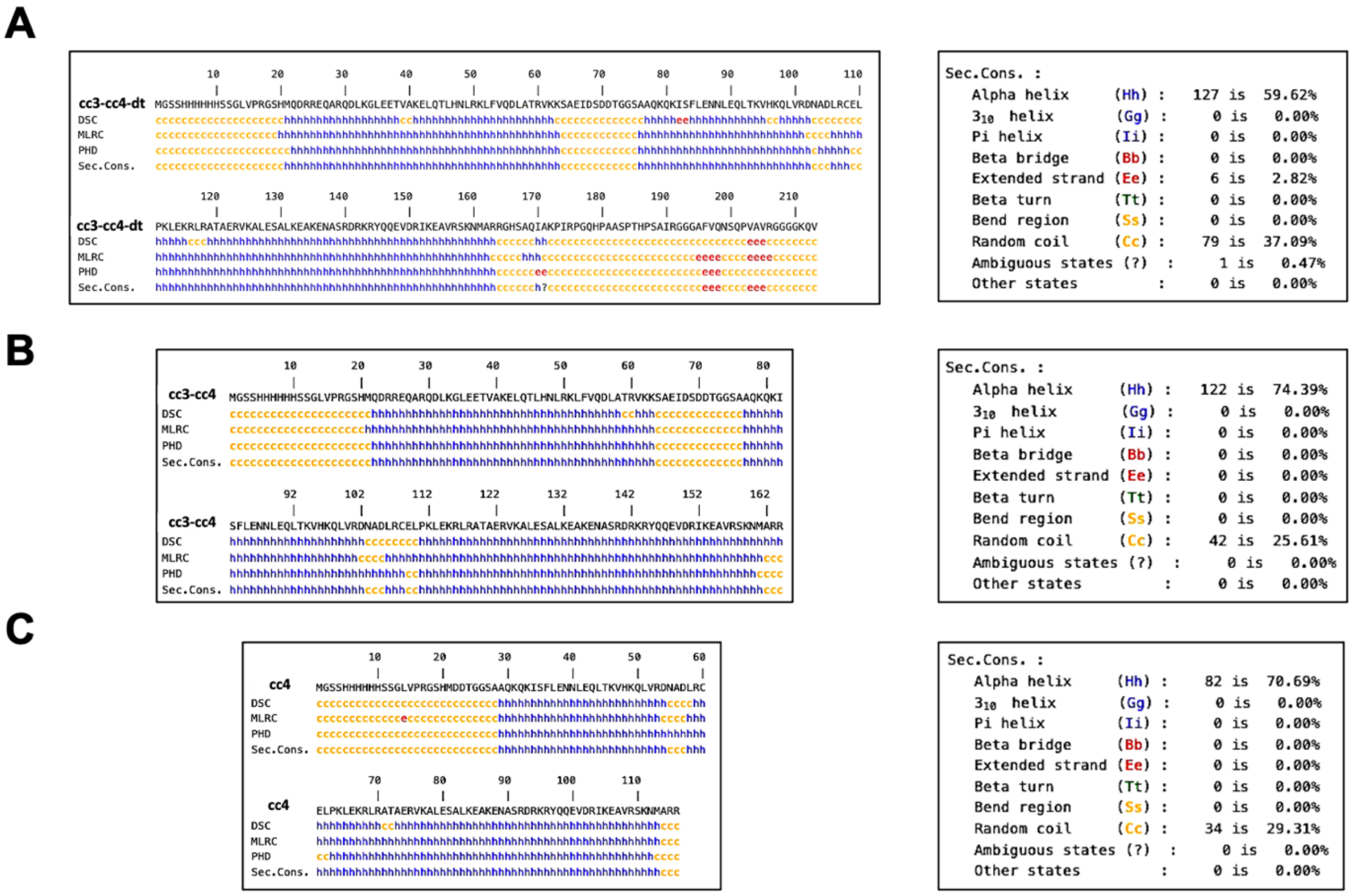

Figure S2. Secondary structure prediction on KIF5B-Tail fragments. Prediction was performed using the software NPS@:Network Protein Sequence Analysis (Combet *et al*, 2000) for each fragment. On the right side the percentage of secondary structures (alpha helix, beta strand and random coil) for each fragment is indicated.

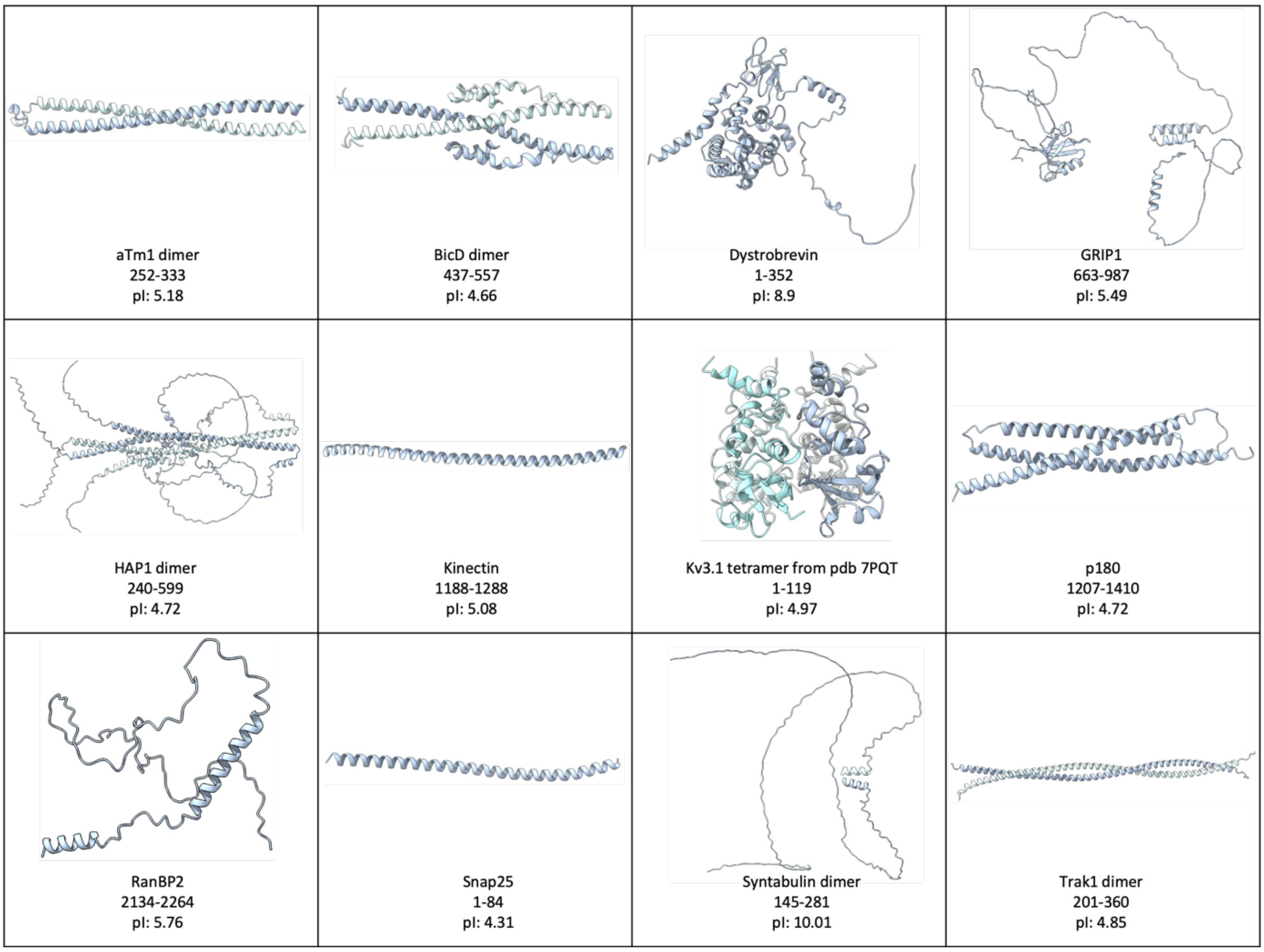

Figure S3. Models of the kinesin binding region of proteins showed to interact with the CC4 domain of the heavy chain of kinesin-1. The models were generated with alpha-Fold multimer for the full protein, except for Kv3.1 which is a crystal structure, but only the proposed kinesin binding region is shown. The residues are indicated below the name of the protein. The confidence of the models is variable mostly because the majority of the proteins are multidomain and have non structured regions; however, the predicted helices have a high confidence. The proteins known to exists as dimers or with predicted heptad repeats able to form dimers are modelled as dimers. Otherwise, the proteins are modelled as monomers. The ability to activate KHC is mentioned when the information has been reported. Figures were generated with ChimeraX.
